# Cul de Sac : An Unusual Presentation of Giant Cell Arteritis

**DOI:** 10.7759/cureus.16361

**Published:** 2021-07-13

**Authors:** Chinenye Osuorji, Naomi Ojumah, Oluwafeyi Adedoyin, Okechukwu Okoye, Ikechukwu Mbonu

**Affiliations:** 1 Internal Medicine, Burrell College of Osteopathic Medicine, Las Cruces, USA; 2 Anatomical Sciences, St. George's University School of Medicine, St. George's, GRD; 3 Internal Medicine, Englewood Hospital and Medical Center, Englewood, USA; 4 Internal Medicine, Newark Beth Israel Medical Center, Newark, USA; 5 Rheumatology, Medical College of Wisconsin, Milwaukee, USA

**Keywords:** giant cell arteritis, large vessel vasculitis, clinical diagnosis, polymyalgia rheumatica, glucocorticoids, temporal artery biopsy, autoimmue

## Abstract

Giant cell arteritis (GCA), previously referred to as temporal arteritis, continues to pose significant diagnostic challenges to clinicians as it could have unusual and atypical presentations. We present the case of a 69-year-old Caucasian male who had presented with painful scrotal swelling and bilateral arm pain and was eventually diagnosed with GCA based on histological findings. His symptoms resolved completely with the initiation of high-dose steroids. It is important to note that some clinical manifestations of GCA could be subtle, atypical, and maybe entirely extracranial. A high index of suspicion is helpful when diagnosing patients who present with non-specific or constitutional symptoms as delay in diagnosis or treatment in these patients could result in severe adverse outcomes.

## Introduction

Giant cell arteritis (GCA) is a chronic granulomatous vasculitis that affects medium and large blood vessels [1]. Major vessels include the aorta and its extracranial branches. These include the external carotid arteries and its tributaries such as the temporal and occipital arteries. Other arteries include the ophthalmic, vertebral, subclavian, axillary, thoracic, and abdominal aorta in some cases. It has an incidence of about 15-25 cases per 100,100 persons and tends to affect older individuals of 50 years and above [1,2].

The clinical manifestation of GCA could be sudden onset or insidious. Most described symptoms include scalp tenderness and headaches in 70-80% of cases, and hearing loss, jaw claudication, and sudden onset of unilateral visual loss in 10-15% of cases [2]. Timely diagnosis and management of GCA is pivotal, especially with ophthalmic involvement, which may result in the most dreaded complication of irreversible vision loss. Preceding events of temporary vision loss have been linked to a greater risk for permanent visual compromise [1,2]. ^ ^In addition, less common complications of episcleritis, scleritis, and extraocular muscular paralysis have been cited [3]. 

Polymyalgia rheumatica is often diagnosed in association with GCA. Non-specific, constitutional symptoms have also been described. These include low-grade fever, malaise, weight loss, anorexia, and generalized fatigue. Although very rare, symptoms suggestive of peripheral neuropathy, strokes, and ischemic complications involving the tongue, scalp, and extremities have also been described. Examination of the temporal region may reveal thickened, cord-like, beaded, diminished, or non-pulsatile vasculature with or without associated tenderness. Careful assessment of other peripheral arterial regions should be assessed and may yield similar findings. Aortitis can be complicated by dissection and aneurysm involving most commonly the thoracic aorta [4-6]. Known urological manifestations of GCA have also been reported as orchitis, epididymitis, as well as testicular mass [7-9]. 

## Case presentation

A 69-year-old man with a history of type 2 diabetes mellitus and hypertension presented to the emergency room with hyperglycemia and complaints of a painful scrotal swelling of four days. He had been riding a snow-mobile for the past week but denied any trauma. He has no history of penile discharge, dysuria, hematuria, and urinary frequency or urgency and reports normal bladder emptying and bowel movement. Vitals were otherwise within normal limits. On physical examination, he was obese with a BMI of 34.8, and had Dupuytren's contracture on the left hand. Eye examination was remarkable for bilateral cataracts. Bilateral hydrocele with tenderness was noted. There was no erythema, warmth, rash, or skin excoriation. The rest of his physical examination was unremarkable.

The patient returned to the emergency room a month later with complaints of worsening dyspnea and generalized weakness. Preliminary pulmonary and cardiac workup was largely unremarkable. However, new-onset thrombocytosis, anemia, and leukocytosis were reported. Findings of CT of the chest, abdomen, and pelvis were not suggestive of any acute or chronic pulmonary process, malignancy, infection, or lymphadenopathy. Laboratory results, however, revealed elevated inflammatory markers with markedly elevated ferritin levels, acute phase reactants, hypoalbuminemia, elevated relic count but unremarkable hemolysis profile. Anti-neutrophil antibody titer, rheumatoid factor, anti-cyclic citrullinated peptide, blood cultures, and Lyme panels were negative.

At this point, he was referred to rheumatology for further evaluation and workup. Despite the lack of cranial symptoms, given the unusual relatively rapid onset of systemic inflammatory symptoms, as well as ruling out most of the other chronic inflammatory conditions, a suspicion of large vessel vasculitis was strongly considered. Other vasculitis syndromes were also ruled out by negative myeloperoxidase and PR-3 anti-neutrophil cytoplasmic antibody serologies. Results of a right temporal artery biopsy revealed intimal thickening and a disrupted internal elastic lamina. There was resolution of the patient's symptoms with high doses of prednisone. He was switched to tocilizumab and symptoms remained controlled. Resultant hyperglycemia was managed adequately with adjustments to his insulin regimen. See Table 1 for summary of lab results. 

 

**Table 1:** Summary of Lab Results

Parameter	Value	Reference value
Hemoglobin	9.4	13.5-17.5 g/L
Hematocrit	30	41-53%
Leukocyte count	17.9	4500-11,000/mm^3^
Erythrocyte count	3.1	4.3-5.9 million/mm^3^
Prothrombin time	6.8	11-15 seconds
Mean corpuscular volume	97	80-100 um^3^
Mean corpuscular hemoglobin	30.3	25-35 pg/cell
Mean corpuscular hemoglobin concentration	31	31-36% Hb/cell
Red cell distribution width	14.7	11.8-14.5%
Platelet count	631,000	150,000-400,000/mm^3^
C-reactive protein	26.1	<3.0
Ferritin	1653	24-336 ug/L
Creatinine	0.9	0.6-1.2 mg/dL
Urea	19	7-18 mg/dL
Sodium	129	136-146 mEq/L
Potassium	4.7	3.5-5.0 mEq/L
Chloride	92	95-105 mEq/L
Bicarbonate	25	22-28 mEq/L
Calcium	9.1	8.4-10.2 mg/dL
Glucose	586	70-99 mg/dL
Albumin	2.8	3.4-5.4 g/dL
Erythrocyte sedimentation rate	>140	0-15 mm/h
Alkaline phosphatase	97	25-100 U/L
Alanine aminotransferase	15	10-40 U/L
Aspartate aminotransferase	24	12-38 U/L
Total bilirubin	0.2	0.1-0.2 mg/dL
Proteinase 3 anti-neutrophil cytoplasmic antibodies	Non-reactive	0-19 AU/mL
Myeloperoxidase antibody	Non-reactive	0-19 AU/mL
Anti-nuclear antibody	0.2	1:80
Rheumatoid factor	18	0-20 IU/mL
Anti-cyclic citrullinated peptide	Non-reactive	<20 u/mL

## Discussion

Pathophysiology 

The pathogenesis of GCA is poorly understood. Activation of immature dendritic cells within the tunica adventitia of the vessels leads to activation and proliferation of CD4^+^ naive T-cells. These cells then differentiate into T helper (Th)1, Th17, and T (Treg) regulatory cells. Macrophages produce metalloproteinases that lead to the destruction of the lamina Intima. A cascade of reactions occur in which reactive oxidation and production of various growth factors, including the release of interleukin (IL)-6, IL-8, and interferon-gamma, lead to vascular inflammation and damage with resultant obliteration and blockage. This is further worsened by dysregulated vascular repair. HLA DR-01 is thought to play a role and thus suggestive of antigen selection and presentation phenomena [10]. 

Diagnosis

Laboratory findings could be non-specific; however, the elevated inflammatory markers erythrocyte sedimentation rate/C-reactive protein, reactive transaminitis, thrombocytosis, and anemia are common in most patients. The utilization of the American College of Rheumatology classification criteria (1990) is helpful in differentiating various other types of vasculitis. The definitive diagnosis of GCA is made via histopathologic findings on temporal arterial biopsy, which is considered the gold standard, and this led us to the diagnosis in this case. Histologic changes include presence of polynucleated giant cells with inflammatory infiltration of the vascular media-intima interspaces, bridging of the internal elastic lamina layer with resultant part or total occlusion of blood supply to the involved region, and subsequent clinical consequences [11,12]. Sensitivity of above 90% has been associated with the use of color duplex ultrasonography as a non-invasive, easily accessible diagnosis tool especially with the revelation of arterial wall swelling (Halo sign). However, results are largely operator dependent and sensitivity is usually obscured in patients with underlying atherosclerosis [13,14]. Although the temporal arterial biopsy was the most useful diagnostic tool in this case, current advances in non-invasive methods of diagnosis via radiographic imaging have helped circumvent the limitations encountered when surgical specimens or biopsy is impractical. However, there remains divergent opinions on timing, tools for evaluation, and defining GCA. MRI, CT, and positron emission tomography (PET) scans have been used to aid the diagnosis of GCA with individually recognized pros and cons. MRI and MR angiography are reportedly very sensitive in visualizing vascular wall edema and also useful when wider sections of vascular involvement are being examined [13]. Evidence of aortitis was reported in 45-65% of patients newly diagnosed with GCA that were evaluated with CTA [14]. Subsequently, CTA studies have also reported aortic structural disruptions seen at the time of diagnosis in 15-23% of the patients. The fluorodeoxyglucose PET scan has been observed to show increased metabolic activity within the large arteries in up to 83% of patients with GCA. There is no PET criterion that currently defines the limits for vascular inflammation and/or prevent misinterpretation of vascular inflammation especially in the older population where vascular uptake could be exaggerated due to the presence of co-existing atherosclerosis [15]. 

Management

Glucocorticoids remain the treatment of choice in GCA, especially given its prompt anti-inflammatory properties that help control symptoms and avert serious adverse ischemic sequela. There are considerations and therapeutic recommendations that favor a shift toward steroid-sparing medications due to well-established risks of complications and adverse outcomes associated with steroid use [16]. Anti-platelet therapy like aspirin may be used to lower the risk of major adverse cardiovascular events [17]. Adjunctive therapies like methotrexate, and IL-6 inhibitors like tocilizumab have demonstrated significant clinical improvement and even sustained remission in most cases [18,19].

## Conclusions

This patient had minimal symptoms indicative of GCA. However, a temporal biopsy revealed the typical findings in GCA. This case report highlights yet another symptomatology of GCA and emphasizes on the importance of biopsy as well as having a high index of suspicion. Diagnosis using PET scan is also a method that needs to be explored especially in older patients with signs of chronic long-standing atherosclerosis as in this patient.
